# Cajan leaf combined with bone marrow-derived mesenchymal stem cells for the treatment of osteonecrosis of the femoral head

**DOI:** 10.3892/etm.2014.1622

**Published:** 2014-03-14

**Authors:** DA SHI, YINDI SUN, JICHAO YIN, XIAOCHEN FAN, HONGHAO DUAN, NA LIU, WEI HE

**Affiliations:** 1 Department of Orthopedics of Traditional Chinese Medicine, Hong Hui Hospital Affiliated to Xi’an Jiaotong University College of Medicine, Xi’an, Shaanxi 710054, P.R. China; 2 Department of Orthopedics, The First Affiliated Hospital, Guangzhou University of Chinese Medicine, Guangzhou, Guangdong 510405, P.R. China

**Keywords:** cajan leaf, mesenchymal stem cell, osteonecrosis of the femoral head, treatment

## Abstract

The aim of the present study was to observe the curative effect of traditional Chinese cajan leaves, combined with administration of bone marrow-derived mesenchymal stem cells (BMSCs), on osteonecrosis of the femoral head (ONFH) in rats and to investigate the underlying mechanisms. A total of 40 rat ONFH models were established through liquid nitrogen freezing and were subsequently divided into groups: A, control; B, treated with cajan leaf; C, treated with BMSCs and D, treated with cajan leaf combined with BMSCs. Samples were obtained 30 days following treatment, and immunohistochemical staining of vascular endothelial growth factor (VEGF) and image analysis were performed. Chondrocytes and vascular endothelial cells were stained as a result of immunohistochemical staining and group D exhibited markedly deeper staining, and a significantly larger number of stained cells, compared with group A. Thus, in the present study, cajan leaf combined with BMSCs was shown to promote VEGF expression and improve ONFH repair.

## Introduction

Osteonecrosis of the femoral head (ONFH) has a high incidence, however, the underlying pathogenesis of ONFH remains unclear. To date, theories of metabolic disturbance, osteoporosis, intraosseous hypertension, intracellular coagulation and cytotoxicity have been hypothesized to explain its pathogenesis, but the underlying pathological change in ONFH is microcirculation disturbance (1,2). Furthermore, the treatment of ONFH remains a controversial challenge as no safe or effective prevention and treatment methods have been identified (3–5).

As molecular biology has developed, bone marrow derived mesenchymal stem cells (BMSCs) have been applied in the treatment of ONFH; this has initiated a novel strategy for the treatment of ONFH and has been identified as effective in preliminary investigations (6,7). However, the application of cajan leaf in combination with BMSCs in the treatment of ONFH has not yet been investigated.

In the present study, the effect of combining traditional Chinese cajan leaf with BMSCs, for the treatment of ONFH, was observed and the underlying mechanisms were analyzed.

## Materials and methods

### Animals

A total of 40 healthy Sprague Dawley male rats (weight, 200±20 g) were purchased from the Laboratory Animal Center of Zhejiang University (Zhejiang, China). This study was conducted in strict accordance with the recommendations in the Guide for the Care and Use of Laboratory Animals of the National Institutes of Health (2011). The animal use protocol was reviewed and approved by the Institutional Animal Care and Use Committee of Xi’an Jiaotong University College of Medicine (Xi’an, China). The study was approved by the ethics committee of the medical ethics committee of Xi’an Jiaotong University, Xi’an, China.

### Grouping

Following three days of adaptive feeding, the rats were randomized into groups of ten: A, control with no treatment; B, treated with cajan leaf; C, treated with BMSCs and D, treated with cajan leaf and BMSCs. The groups were subject to left-sided ONFH modeling.

### Modeling and handling

Modeling was performed via liquid nitrogen freezing. Following administration of an intraperitoneal anesthesia with 1% pentobarbital sodium (45 mg/kg), each rat was fixed supinely to the surgical table. The skin was prepared and disinfected three times. An anterior longitudinal incision was performed, to cut open the skin, and the muscles were detached to expose the joint capsule and femur head, ensuring that the femoral artery was not damaged. Half of the femur head was dislocated out of the acetabulum, the femur head was drilled using a rat puncture needle and the surrounding tissues were protected using sterile gauze. Subsequently, the front of the femoral head was punctured 10 times (for 20 secs each time) using the metal bar in the nitrogen canister. Subsequent to heating with warm physiological saline, groups C and D were injected with BMSCs. BMSCs were obtained from the femur and the shinbone of Sprague Dawley rats, which were cultured in Dulbecco’s Modified Eagle Medium (DMEM) containing 10% fetal bovine serum, 100 μ/ml penicillin and 100 μ/ml streptomycin. Next, ~5 μl BMSCs (containing ~10^5^ cells) were injected using a microinjector. The incision was sealed using gelatin and sutured layer by layer. The muscular layer was sutured at first, and then the skin was sutured. The rats were injected with penicillin for three consecutive days to prevent infection following the modeling. No other treatment was administered and the rats were caged separately. Administration of cajan leaf commenced one day following the cell injection. An aqueous solution of the cajan leaf was prepared (Beijing Normal University, Beijing, China) and injected locally into the hip joint on the side that was used for preparing the model. A dose of 40 mg/kg cajan leaf was administered in an injection volume of 0.4 ml per rat; groups A and C received an equal volume of physiological saline. Each rat was injected once per day for 30 consecutive days.

### Sample collection

Ten rats from each group were sacrificed 30 days following treatment. The integral femoral heads were obtained under aseptic conditions, wrapped with wet gauze and stored in a low temperature refrigerator.

### Decalcified bone sample preparation

The whole femoral head was fixed in 4% formaldehyde solution for 72 h, which was subsequently decalcified in 10% edetic acid solution for ~14 days (the decalcifying fluid was changed every 7 days). Histomorphological observation and immunohistochemical staining were performed until the cancellous bone was pierceable with a pin.

### Histomorphological observation

Segments of the decalcified bone samples were dehydrated using a gradient of ethanol, wax-dipped, embedded in paraffin and cut into 4- μm sections. One section was subject to hematoxylin and eosin staining and the other section was used for immunohistochemical staining of vascular endothelial growth factor (VEGF) and image analysis.

### Immunohistochemical staining and image analysis

The sections were loaded on to polylysine pretreated microscopic slides and placed in a 60°C oven for 30 min to increase adhesion. They were routinely dewaxed and placed in a 3% H_2_O_2_ solution for 10 min at room temperature, to block endogenous peroxidase activity. Subsequently, the sections were rinsed three times in distilled water, soaked in 0.01 M citrate buffer and heated to boiling point, at which point the power was immediately cut off. After 10 min, the heating process was repeated and the sections were reacted with antigen retrieval buffer for 5–10 min to expose additional antigens. The sections were rinsed three times and incubated with normal goat serum confining liquid for 20 min. Redundant liquid was removed and the sections were incubated overnight at 4°C with a 1:150 dilution of rabbit anti-mouse VEGF antibody (Sigma, St. Louis, MO, USA). Phosphate-buffered saline (PBS) was used to wash the sections, which were then incubated at 37°C, with biotinylated goat anti-rabbit IgG. Subsequent to this, sections were incubated with streptavidin-biotin complex for 20 min. The sections were washed four times with PBS and stained with 3,3′-diaminobenzidine solution at room temperature, rinsed with distilled water, counterstained with hematoxylin, dehydrated, cleared, mounted and observed under a microscope (Olympus, Tokyo, Japan). The cells exhibiting homogeneously buffy-stained cytoplasm and membranes were considered to exhibit positive expression.

Following staining, the sections were observed under a light microscope (magnification, ×200). Visual fields from each section were randomly selected to determine positive expression. Positive expression of VEGF at the broken ends of the fractured bones was determined using mean gray scale values and compared using an image analysis system (Nikon ACT-1U, Nikon Corporation, Tokyo, Japan). The gray scales represented the signal intensity within the cells, whereby a reduced gray scale indicated a higher intensity. Five visual fields were randomly selected, total cells and positive cells were counted from which the positive cell percentage was calculated.

### Statistical analysis

All data were analyzed using SPSS software (SPSS Inc., Chicago, IL, USA). Enumeration data are presented as mean ± standard deviation.

## Results

### Histomorphological changes

**Figure 1:**
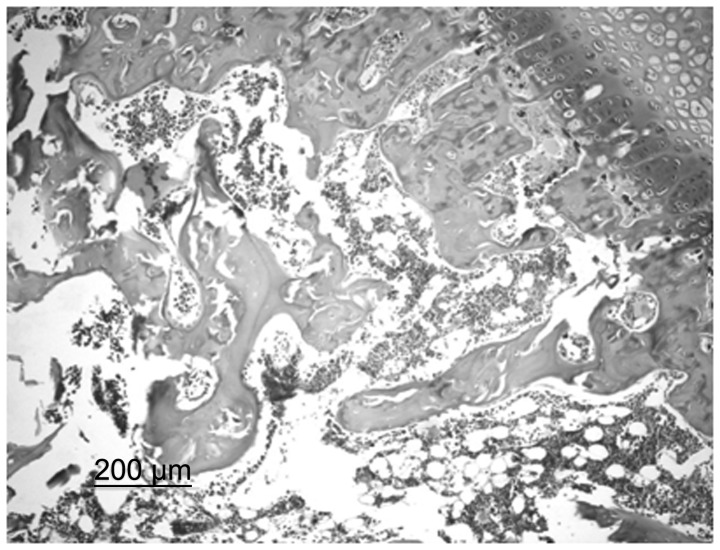
Control group; the bone trabeculae were sparse, thin and exhibited ruptures. Structural disorder and bone fragments were apparent (magnification, ×10; hematoxylin-eosin staining).

**Figure 2:**
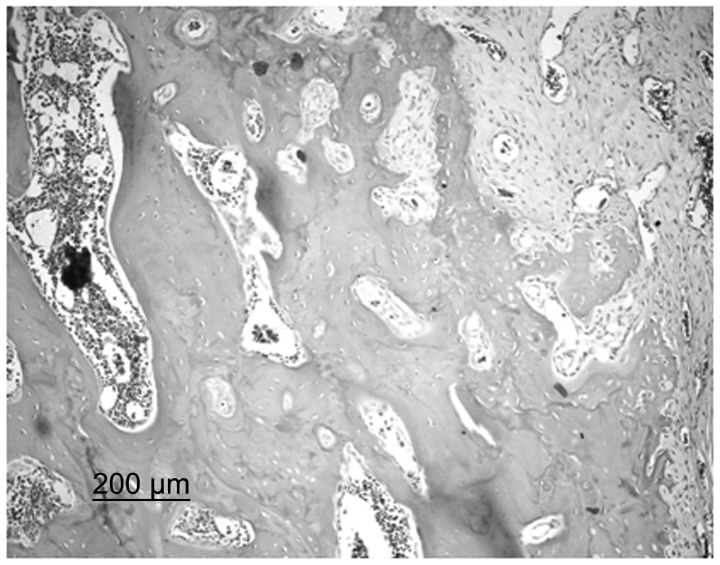
Cajan leaf + BMSC group; the bone trabeculae exhibited a regular alignment, which were dense and full without ruptures. A large quantity of hematopoietic cells were apparent in the pulp chamber and no large fat drops were observed. The number of spindle-shaped osteoblasts, in a dense alignment along the margins of the bone trabeculae, increased (magnification, ×10; hematoxylin-eosin staining). BMSC, bone marrow derived mesenchymal stem cells.

In the control group, bone trabeculae were sparse, thin and exhibited ruptures. Structural disorder and bone fragments were apparent. Osteocytes in the bone trabeculae displayed pyknosis or margination and in specific cases, osteocytes were not present. In addition, the number of empty bone lacunae markedly increased, the volume of the lipocytes in the pulp chamber increased and specific lipocytes fused into a bubble shape. Spindle-shaped osteoblasts were observed along the margins of the bone trabeculae in small numbers (Fig. 1). In the BMSC group, bone trabeculae were incomplete and, although their alignment was ordered, a small number were broken. It was possible to observe the nuclei of osteocytes on the bone trabeculae and a number of empty bone lacunae were present. A large quantity of spindle-shaped osteoblasts were observed along the margins of the bone trabeculae. In the cajan leaf and cajan leaf + BMSC groups, bone trabeculae exhibited a regular alignment. Trabeculae were dense and full without ruptures and the nuclei of osteocytes were clearly observed. In addition, the presence of empty bone lacunae was rare, hematopoietic cells were abundant in the pulp chamber and no large fat drops were observed. Furthermore, the quantity of spindle-shaped osteoblasts in a dense alignment along the margins of the bone trabeculae increased (Fig. 2).

### Immunohistochemical staining

**Figure 3:**
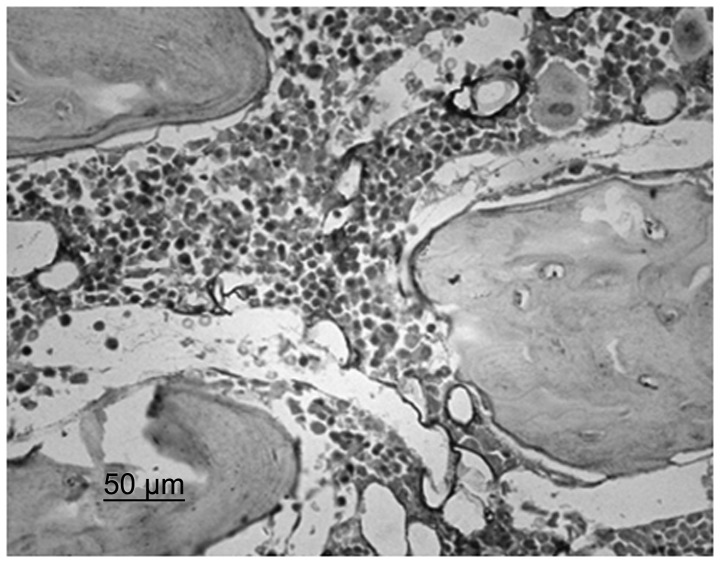
Positive expression of VEGF in the cajan leaf + BMSC group as determine by immunhistochemical staining using an antibody against VEGF (magnification, ×40). VEGF, vascular endothelial growth factor; BMSC, bone marrow derived mesenchymal stem cells.

**Figure 4:**
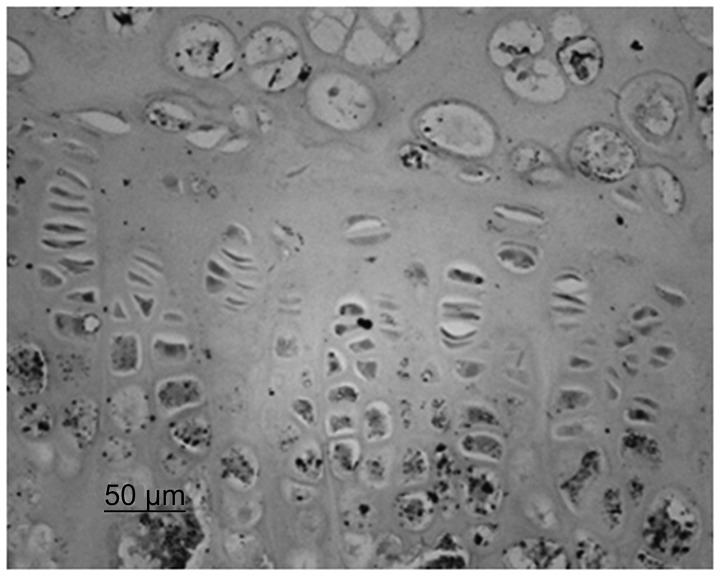
Positive expression of chondrocytes with deep staining in the cajan leaf group (magnification, ×40; Immunohistochemical staining).

Immunohistochemistry identified that chondrocytes and VEGF were stained. Although immature and mature chondrocytes in the chondrogenic zone were stained, the staining of the mature chondrocytes was deeper. Compared with the control group, the cajan leaf and cajan leaf + BMSC groups exhibited deeper staining of chondrocytes and VEGF, and a greater quantity of positively expressed cells (Figs. 3 and 4).

### Image analysis

The gray scale assays identified that the positive expression intensity of groups B–D significantly increased compared with that of group A (P<0.01). Although no significant difference was observed between groups B and D (P>0.05), group D exhibited a greater tendency towards a positive expression intensity. The results showed that the positive expression rates of groups B–D were markedly higher than the rates of group A (P<0.01). Furthermore, groups A–C showed significant differences in positive expression compared with group D (P<0.01). The results are summarized in Table I.

## Discussion

The primary pathological change in ONFH is the obstruction of intraosseous blood supply, which leads to a disturbance in the microcirculation of the femoral head. Although the exact incidence rate of ONFH remains unknown, 1,000–2,000 patients are diagnosed with this condition annually in the United States (8). The treatment of ONFH is complex and generally classified into surgical and non-surgical treatment methods; however, the curative effects of the two methods vary (9–13). Li and Wang (14) observed the effect of *Epimedium brevicornum* on hormonal ONFH and identified that its effect may be correlated with the improvement of local blood circulation in the femoral head and the promotion of the proliferation, differentiation and maturation of osteoblasts *in vitro*. These observations highlighted the possibility of administering cajan leaf for the treatment of ONFH.

Bone tissue engineering is a novel branch of scientific research, which aims to design, construct, culture and maintain living cells, to study biological substitutes, repair and reconstruct the structure of human tissues and organs, and to maintain or improve their functions based on the principles and techniques of biology and engineering. The key components of tissue engineering may be summarized as stent materials, seed cells and signal factors. BMSCs are non-hematopoietic stem cells in the bone marrow, which support and regulate hematogenesis *in vivo*, as well as *in vitro*. BMSCs are distributed in a variety of tissues and organs *in vivo* and have a multi-directional differentiation potential, which enables them to differentiate into osteoblasts, fibroblasts, reticulocytes, lipocytes and endothelial cells; for these reasons, BMSCs are currently being extensively studied. In addition, the gradual development of the technique of inducing the differentiation of BMSCs towards osteoblasts, indicates a general trend towards the utilization of BMSCs for treatment of ONFH. Although BMSCs have a certain curative effect on ONFH (7,15,16), the underlying mechanisms have not been elucidated. Therefore, the present study aimed to identify the possible mechanisms underlying the curative effect of BMSCs on ONFH, based on the hypothesis that BMSCs improve ONFH repair by promoting revascularization.

The regeneration and repair of ONFH is a complex physiological and biochemical process, which is accompanied by vascularization. VEGF specifically acts on endothelial cells to promote their proliferation and vascularization, thereby participating in bone regeneration and repair. When using bone grafts in clinical practice to repair defects, a sufficient blood supply to the bone graft bed is required. In autogenous bone implantation, a vascular pedicle bone graft or a graft with muscular flaps is frequently used to increase blood supply in order to promote the early survival of the bone graft. Therefore, for bone formation, an optimal vascular net is necessary to provide nutrition and oxygen, transport osteogenic precursor cells and secrete growth factors, which are required by the osteoblasts (17,18). In the present study, the results showed that treatment with cajan leaf, BMSCs alone and cajan leaf + BMSCs were all capable of locally stimulating a high expression of VEGF, especially in the cajan leaf and cajan leaf + BMSCs groups. The mechanisms underlying the repair-promoting effect of cajan leaf + BMSCs on ONFH may, therefore, be as follows. Although local hypoxia following ONFH stimulated the expression of VEGF, cajan leaf combined with BMSCs increased VEGF expression. Such an increase strengthened the vascular proliferation in the necrotic area and formed a complete vascular net. Accordingly, the formation of this net provided increased nutrition and oxygen for the necrotic area, which conveyed increased osteogenic precursor cells and secreted related growth factors, thereby accelerating ONFH repair.

As investigation of ONFH continues, the analysis of genes associated with this condition has gained increasing attention worldwide (19–23). Although further progress in the investigation of BMSCs, as well as the application of BMSCs in the treatment of ONFH has been made, the study of cajan leaf combined with BMSCs is in its infancy. Furthermore, the underlying mechanisms remain unknown; however, previous studies have laid the foundations and provided a direction for further investigation.

In conclusion, the application of traditional Chinese medicine in the treatment of orthopedic disorders has a long history and has achieved a marked curative effect. Therefore, analyzing traditional Chinese medicine using modern technology whilst continuing to link traditional Chinese medicine with gene research, may provide a direction for future investigation.

## Figures and Tables

**Table I:** Positive expression rate and gray scale of each group.

Group	Positive expression rate, %	Gray scale
Control	22.1323±2.6852^b^	140.7500±2.5495^b^
Cajan leaf	40.0572±5.2344^a^	125.1250±3.6815^a^^b^
BMSC alone	30.8064±2.2825^a^^b^	135.125±3.1820^a^^b^
Cajan leaf + BMSC	47.0581±4.8905^b^	124.0000±4.7509^a^

a P<0.01, vs. control group;

b P<0.01, vs. cajan leaf + BMSC group.

BMSC, bone marrow derived mesenchymal stem cells.

## References

[1] Assouline-Dayan Y, Chang C, Greenspan A, Shoenfeld Y, Gershwin ME (2002). Pathogenesis and natural history of osteonecrosis. Semin Arthritis Rheum.

[2] Aaron RK (2001). Concepts of the pathogenesis of osteonecrosis. Tech Orthop.

[3] Ivankovich DA, Rosenberg AG, Malamis A, Aaron RK (2001). Reconstructive options for osteonecrosis of the femoral head. Tech Orthop.

[4] Jones LC, Hungerford DS (2003). Overview of osteonecrosis of the hip and current treatment options. Curr Opin Orthop.

[5] Zhao DW, Hu YC (2012). Chinese experts’ consensus on the diagnosis and treatment of osteonecrosis of the femoral head in adults. Orthop Surg.

[6] Zhao D, Cui D, Wang B (2012). Treatment of early stage osteonecrosis of the femoral head with autologous implantation of bone marrow-derived and cultured mesenchymal stem cells. Bone.

[7] Gangji V, De Maertelaer V, Hauzeur JP (2011). Autologous bone marrow cell implantation in the treatment of non-traumatic osteonecrosis of the femoral head: Five year follow-up of a prospective controlled study. Bone.

[8] Babis GC, Sakellariou V, Parvizi J, Soucacos P (2011). Osteonecrosis of the femoral head. Orthopedics.

[9] Baksi DP, Pal AK, Baksi DD (2009). Long-term results of decompression and muscle-pedicle bone grafting for osteonecrosis of the femoral head. Int Orthop.

[10] Marker DR, Seyler TM, McGrath MS, Delanois RE, Ulrich SD, Mont MA (2008). Treatment of early stage osteonecrosis of the femoral head. J Bone Joint Surg Am.

[11] Nozawa M, Maezawa K, Matsuda K (2008). Rotational acetabular osteotomy for osteonecrosis of the femoral head after intracapsular fracture of the neck of the femur. J Orthop Trauma.

[12] Wei BF, Ge XH (2011). Treatment of osteonecrosis of the femoral head with core decompression and bone grafting. Hip Int.

[13] Malizos KN, Karantanas AH, Varitimidis SE, Dailiana ZH, Bargiotas K, Maris T (2007). Osteonecrosis of the femoral head: etiology, imaging and treatment. Eur J Radiol.

[14] Li HY, Wang YS (2012). Effect of epimedium on blood rheology and bone density of rats with steroid-induced femoral head necrosis. Chin J Exp Surg.

[15] Yamasaki T, Yasunaga Y, Ishikawa M, Hamaki T, Ochi M (2010). Bone-marrow-derived mononuclear cells with a porous hydroxyapatite scaffold for the treatment of osteonecrosis of the femoral head: a preliminary study. J Bone Joint Surg Br.

[16] Yoshioka T, Mishima H, Akaogi H, Sakai S, Li M, Ochiai N (2011). Concentrated autologous bone marrow aspirate transplantation treatment for corticosteroid-induced osteonecrosis of the femoral head in systemic lupus erythematosus. Int Orthop.

[17] Liu B, Cao Y, Wang D, Yao G, Bi Z (2012). Vascular endothelial growth factor -634G/C polymorphism associated with osteonecrosis of the femoral head in a Chinese population. Genet Test Mol Biomarkers.

[18] Hang D, Wang Q, Guo C, Chen Z, Yan Z (2012). Treatment of osteonecrosis of the femoral head with VEGF165 transgenic bone marrow mesenchymal stem cells in mongrel dogs. Cells Tissues Organs.

[19] Hong JM, Kim TH, Kim HJ, Park EK, Yang EK, Kim SY (2010). Genetic association of angiogenesis- and hypoxia-related gene polymorphisms with osteonecrosis of the femoral head. Exp Mol Med.

[20] Kim TH, Hong JM, Kim HJ, Park EK, Kim SY (2010). Lack of association of MTHFR gene polymorphisms with the risk of osteonecrosis of the femoral head in a Korean population. Mol Cells.

[21] Lee HJ, Choi SJ, Hong JM (2009). Association of a polymorphism in the intron 7 of the SREBF1 gene with osteonecrosis of the femoral head in Koreans. Ann Hum Genet.

[22] Tateda K, Okazaki S, Nagoya S (2012). The suppression of TRIM21 and the accumulation of IFN-α play crucial roles in the pathogenesis of osteonecrosis of the femoral head. Lab Invest.

[23] Tang TT, Lu B, Yue B (2007). Treatment of osteonecrosis of the femoral head with hBMP-2-gene-modified tissue-engineered bone in goats. J Bone Joint Surg Br.

